# Designing Magnetic Topological Insulator Trilayers for Highly Efficient Spin–Orbit Torque Switching

**DOI:** 10.1021/acs.nanolett.6c02154

**Published:** 2026-06-04

**Authors:** Ling-Jie Zhou, Deyi Zhuo, Han Tay, Zi-Jie Yan, Pu Xiao, Xiaoda Liu, Bomin Zhang, Cui-Zu Chang

**Affiliations:** Department of Physics, 8082 The Pennsylvania State University, University Park, Pennsylvania 16802, United States

**Keywords:** Magnetic topological insulator, heterostructure design, spin−orbit torque switching, edge-current chirality, chemical potential asymmetry

## Abstract

Spin–orbit torque (SOT) enables efficient electrical control of magnetization, offering a pathway toward low-power spintronic devices. Magnetic topological insulators (TIs), with spin-momentum-locked surface states and intrinsic ferromagnetism, provide a unique platform for switching the edge-current chirality in quantum anomalous Hall (QAH) insulators. Here, we employ molecular beam epitaxy to synthesize a series of magnetic TI trilayers with controlled layer thicknesses on heat-treated SrTiO_3_(111) substrates. Electrical transport measurements reveal that SOT-driven magnetization reversal and the associated switching of QAH edge current chirality are governed by a SrTiO_3_(111) substrate-induced charging effect, which generates a chemical potential asymmetry between the top and bottom magnetic TI layers. The switching polarity and efficiency are further tuned through heterostructure design, gate voltage, and an in-plane magnetic field. These findings identify chemical-potential asymmetry as the key mechanism for achieving a large SOT switching ratio and establish a route toward electrical control of edge current and QAH-based logic and memory devices.

## Graphical abstract

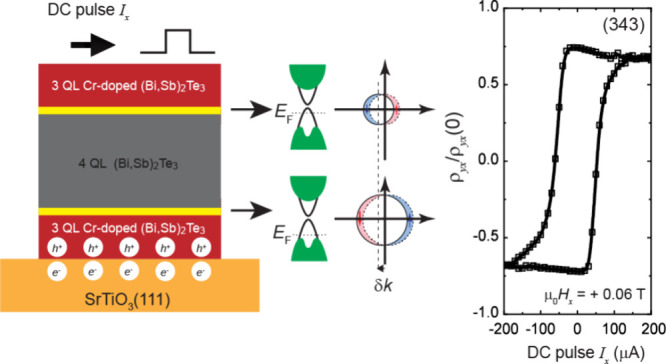

The electrical control of magnetic states has long been a central goal in condensed-matter physics and spintronics.^1–6^ Magnetic materials are fundamental to spin-based information technologies, such as magnetic random-access memory (MRAM), in which data bits are stored by aligning two ferromagnetic layers separated by a tunneling barrier.^7^ Spin-transfer torque (STT) has been used to switch magnetization by injecting spin-polarized currents through an insulating barrier,^8–11^ but the high current densities required for STT can limit device endurance. However, spin–orbit torque (SOT) offers an alternative route by using spin–orbit coupling (SOC) in adjacent nonmagnetic layers to generate spin accumulation via the spin Hall or Rashba-Edelstein effects.^1–3,12–14^ This mechanism enables magnetization switching without passing current through the insulating barrier, greatly enhancing the energy efficiency and device reliability.^1,15^ Therefore, SOT has become a cornerstone for developing next-generation nonvolatile memories and logic devices, stimulating the intensive exploration of new materials that can serve as highly efficient spin sources.

A wide range of materials has been explored for SOT-based devices, including heavy metals,^13,14,16^ topological insulators (TIs),^17–21^ and transition-metal dichalcogenides.^22,23^ Among them, TIs have emerged as particularly promising because their Dirac surface states possess spin-momentum locking, which intrinsically links charge and spin currents.^24–26^ When doped with transition-metal elements such as Cr and/or V, TIs exhibit long-range ferromagnetism with perpendicular magnetic anisotropy,^27–31^ an essential feature for scalable MRAM architectures. The breaking of time-reversal symmetry in these magnetic TIs opens a magnetic exchange gap at the Dirac point, giving rise to the quantum anomalous Hall (QAH) insulators with dissipation-free chiral edge channels.^28–30,32–34^ The interplay between magnetism and chiral edge currents enables the electrical control of edge chirality through SOT-induced magnetization reversal. Our recent experiments demonstrated current-driven switching of edge-current chirality in magnetic TI trilayers with the QAH state,^6^ marking a major step toward realizing electrically controllable QAH devices. However, the microscopic mechanisms underlying SOT-driven switching of edge-current chirality in QAH insulators remain elusive. In magnetic TI trilayers, current injection typically induces opposite spin accumulations at the top and bottom surfaces, canceling the total SOT and yielding a vanishing switching ratio. In contrast, our experimental results reveal substantial magnetic domain reversal, leading to SOT switching of the edge-current chirality in QAH insulators.^6^

In this work, we systematically investigate the microscopic origin of SOT-driven magnetization switching in magnetic TI trilayers. Using molecular beam epitaxy (MBE), we fabricated a series of high-quality magnetic TI trilayers with precisely controlled layer thicknesses. Through electrical transport measurements, we reveal that magnetization reversal and the associated edge current chirality switching are governed by a substrate-induced charging effect. This effect, arising from the SrTiO_3_(111) substrate,^27–29,35^ creates an asymmetric chemical-potential alignment between the top and bottom magnetic TI layers, leading to unequal spin accumulation and markedly different SOT efficiencies. We further demonstrate that the switching polarity and magnitude can be tuned via heterostructure design, gate voltage, and an in-plane magnetic field, consistent with SOT symmetry. These findings identify chemical potential asymmetry as the key mechanism underlying highly efficient SOT switching and provide direct evidence for the controllable coupling between ferromagnetism and topological surface states in QAH insulators.

All magnetic TI trilayers used in this work are grown on heat-treated SrTiO_3_(111) substrates in a commercial MBE chamber (Omicron Lab 10) with a base pressure below 2 × 10^–10^ mbar. To examine the influence of the substrate, control trilayer samples are also grown on InP(111)­A substrates. Each trilayer sample consists of *m* quintuple layer (QL) Cr-doped (Bi,Sb)_2_Te_3_ /4 QL (Bi,Sb)_2_Te_3_/*n* QL Cr-doped (Bi,Sb)_2_Te_3_, denoted as the (*m*4*n*) heterostructure (Figure 1a). The Cr dopants induce strong out-of-plane ferromagnetism while simultaneously decreasing the SOC. With heavy Cr doping, the Cr-doped (Bi,Sb)_2_Te_3_ layer is a trivial ferromagnetic insulator,^36–38^ so the middle undoped (Bi,Sb)_2_Te_3_ spacer layer hosts topological surface states across the Cr-doped (Bi,Sb)_2_Te_3_ /(Bi,Sb)_2_Te_3_ interfaces. The ferromagnetic order in Cr-doped (Bi,Sb)_2_Te_3_ layers breaks time-reversal symmetry and opens a magnetic exchange gap at the Dirac point, giving rise to the chiral edge channel and the QAH effect (Figure 1a).^28–30,32–34^ The Bi/Sb ratio is optimized to set the chemical potential of the entire trilayer near the charge-neutral point, and a back gate is used to finely tune the carrier density. When either *m* or *n* is zero, the trilayer structure reduces to a bilayer, in which one surface hosts a gapless Dirac cone, whereas the other hosts gapped Dirac surface states (Figure 1b,c). All Hall bar devices with a width *w* of ∼ 2 μm and an aspect ratio *l*/*w* of ∼ 4 are fabricated using a two-step electron-beam lithography and argon-ion milling.^6,39^ Electrical transport measurements are performed in a Physical Property Measurement System (PPMS, Quantum Design DynaCool, 1.7 K, 9 T). More details on MBE growth, device fabrication, and electrical transport measurements are provided in the Supporting Information.

**Figure 1:**
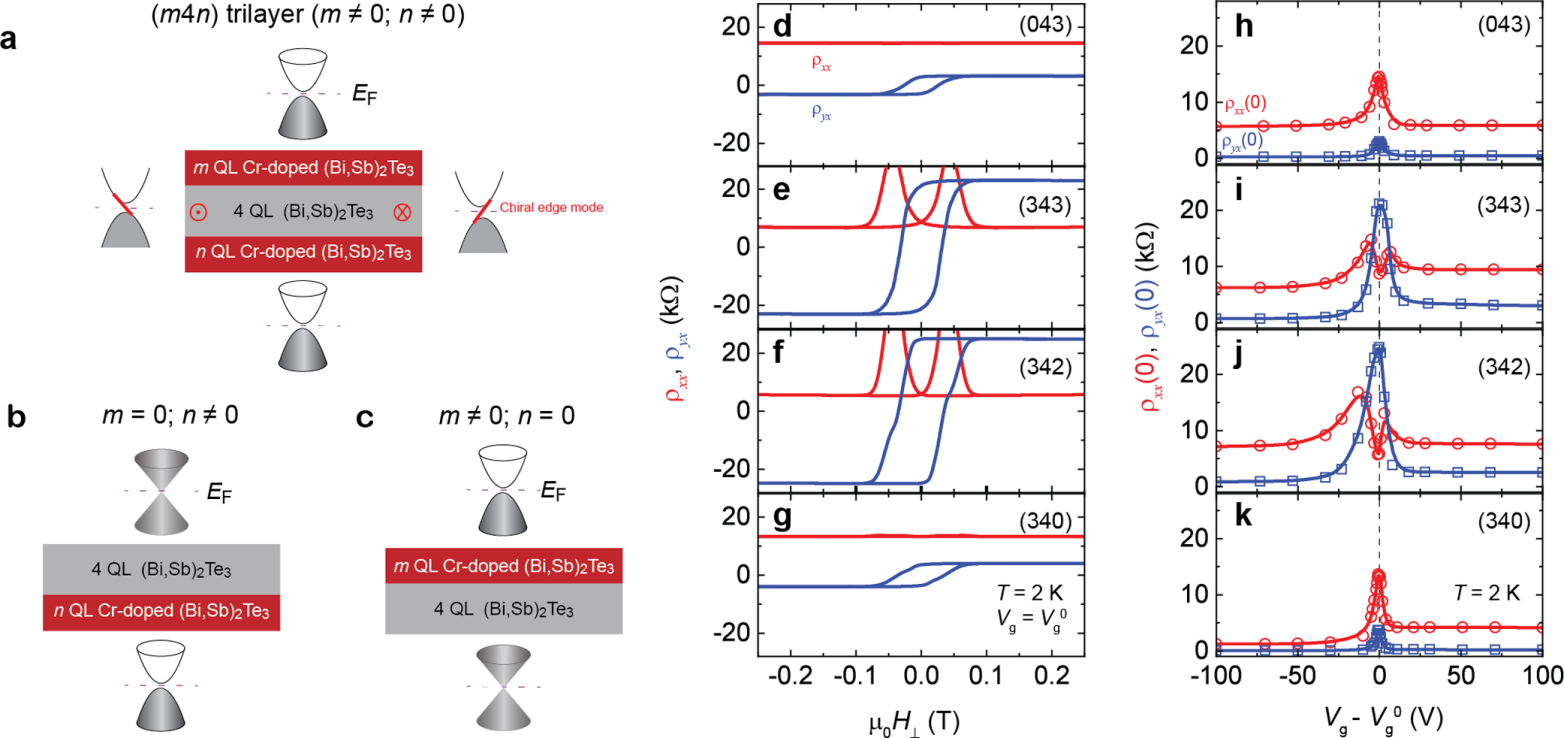
**Transport results of magnetic TI (** ***m*** **4** ***n*** **) trilayers. a**, Schematic of a (*m*4*n*) trilayer structure, consisting of *m* QL Cr-doped (Bi,Sb)_2_Te_3_/4 QL (Bi,Sb)_2_Te_3_/*n* QL Cr-doped (Bi,Sb)_2_Te_3_. The two Cr-doped (Bi,Sb)_2_Te_3_ layers open magnetic exchange gaps in the top and bottom Dirac surface states, while the chiral edge current circulates along the sample edges. **b**, Schematic of the bilayer structure for *m* = 0. **c**, Schematic of the bilayer structure for *n* = 0. **d-g**, μ_0_ *H*_⊥_-dependent *ρ*_*xx*_ (red) and *ρ*_*yx*_ (blue) of the (043) (**d**), (343) (**e**), (342) (**f**), and (340) (**g**) heterostructures at *V*_g_ = *V*_g_^0^ and *T* = 2 K. **h-k**, (*V*_g_ – *V*_g_^0^)-dependent *ρ*_*xx*_(0) (red circles) and *ρ*_*yx*_(0) (blue squares) of the (043) (**h**), (343) (**i**), (342) (**j**), and (340) (**k**) heterostructures at μ_0_ *H*_⊥_ = 0 T and *T* = 2 K. The values of *V*_g_^0^ are +1 V, +5 V, +2 V, and −1 V for the (043), (343), (342), and (340) heterostructures, respectively.

To investigate the SOT switching mechanism in magnetic TI trilayers, we employ MBE to fabricate a series of magnetic TI samples with varied layer thicknesses, specifically the (043), (343), (342), and (340) heterostructures. Our prior studies^30,36,40^ have shown that the (343) and (342) heterostructures typically exhibit the highest-quality QAH effect. To separately investigate the SOT contributions generated by the top and bottom topological surfaces, we also fabricate the (043) and (340) heterostructures, in which one of the two heavily Cr-doped (Bi,Sb)_2_Te_3_ layers is removed. We first perform magnetotransport measurements to characterize the sample quality at *T* = 2 K (Figure 1d-g). At the charge-neutral point, i.e., *V*_g_ = *V*_g_^0^, the zero-magnetic-field Hall resistance ρ_*yx*_(0) is ∼ 2.972 kΩ, ∼ 21.148 kΩ, ∼ 24.846 kΩ, and ∼ 3.639 kΩ for the (043), (343), (342), and (340) heterostructures, respectively. The corresponding zero-magnetic-field longitudinal resistance ρ_*xx*_(0) is ∼ 14.521 kΩ, ∼ 8.690 kΩ, ∼ 5.824 kΩ, and ∼ 13.384 kΩ for the (043), (343), (342), and (340) heterostructures. The two trilayer samples, i.e., the (343) and (342) heterostructures, exhibit nearly quantized Hall resistance, with ρ_*yx*_(0) reaching 0.819 *h*/*e* ^2^ and 0.963 *h*/*e* ^2^ and corresponding Hall angle α of ∼ 67.6° and ∼ 76.8°, respectively, confirming the appearance of the QAH state at *T* = 2 K in these two trilayer samples.^30^

In contrast, the two bilayer samples, i.e., the (043) and (340) heterostructures, exhibit much smaller ρ_*yx*_(0) of ∼ 0.115 *h*/*e* ^2^ and ∼ 0.141 *h*/*e* ^2^, with corresponding Hall angle α of ∼ 11.3° and ∼ 15.1°, respectively. The significantly reduced ρ_*yx*_(0) and α are attributed to the presence of gapless Dirac surface states on one of the surfaces in the bilayer samples (Figure 1b,c).^41^ We note that for the (342) trilayer, a subtle kink is observed near the coercive field (Figure 1f), presumably due to reduced perpendicular magnetic anisotropy caused by the thinner bottom Cr-doped (Bi,Sb)_2_Te_3_ layer. The appearance of the QAH state in the (343) and (342) trilayers is further confirmed by the (*V*_g_ – *V*_g_^0^)-dependent ρ_*yx*_(0) and ρ_*xx*_(0) behaviors. Specifically, ρ_*yx*_(0) exhibits a peak approaching *h*/*e* ^2^. At the same time, ρ_*xx*_(0) shows a dip near *V*_g_ = *V*_g_^0^ (Figure 1i,j). However, the (043) and (340) bilayers exhibit pronounced peaks in both ρ_*yx*_(0) and ρ_*xx*_(0) near *V*_g_ = *V*_g_^0^, indicating the absence of the QAH state in these two bilayer samples (Figure 1h,k).

Next, we examine the SOT-induced magnetization switching in these four heterostructures. Figure 2a shows a schematic of the SOT-induced magnetization switching in a magnetic TI trilayer. An in-plane magnetic field μ_0_ *H*_*x*_ is applied along the current direction. As noted above, the top and bottom heavily Cr-doped (Bi,Sb)_2_Te_3_ layers are trivial ferromagnetic insulators, leading to the formation of topological surface states at the top and bottom Cr-doped (Bi,Sb)_2_Te_3_/(Bi,Sb)_2_Te_3_ interfaces.^30,36,37^ By injecting a current pulse along the *x*-axis, the Rashba-Edelstein effect induced by the helical Dirac surface states leads to the accumulation of spin ***S*_*y*_** along the *y*-axis at both the top and bottom Cr-doped (Bi,Sb)_2_Te_3_/(Bi,Sb)_2_Te_3_ interfaces.^1,17,20,42^ This spin accumulation exerts both a damping-like torque **τ**_**DL**_ ∝ ***M*** × (***M*** × ***S*_*y*_**) = ***M*** × μ_0_ ***H***_**DL**_ and a field-like torque **τ**_**FL**_ ∝ ***M*** × ***S*_*y*_** = ***M*** × μ_0_ ***H***_**FL**_. Here, μ_0_ ***H*_DL_** and μ_0_ ***H***_**FL**_ denote the effective magnetic fields generated by the damping-like and field-like torques, respectively, with μ_0_ ***H***_**DL**_ ∝ ***M*** × ***S*_*y*_** and μ_0_ ***H***_**FL**_ ∝ ***S*_*y*_**. μ_0_ ***H***_**DL**_ is oriented along the *x*-axis and changes sign upon magnetization reversal, whereas μ_0_ ***H***_**FL**_ is oriented along the *y*-axis and remains even with respect to ***M***. Therefore, to achieve SOT-induced magnetization switching, an external magnetic field along the *x*-axis, μ_0_ *H*_*x*_, is required.^1,13^ Moreover, the opposite helicities of the topological surface states at the top and bottom Cr-doped (Bi,Sb)_2_Te_3_/(Bi,Sb)_2_Te_3_ interfaces generate opposite spin accumulations, resulting in reversed torques and opposite switching directions.

**Figure 2:**
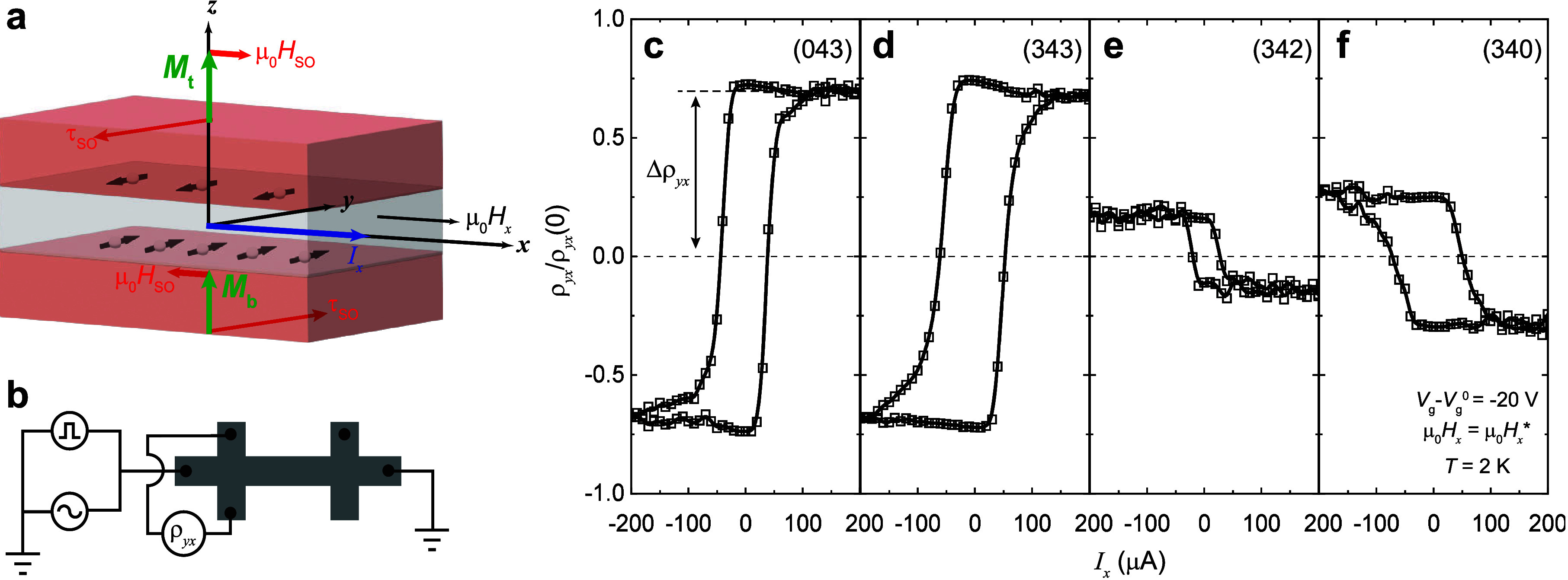
**SOT switching in magnetic TI (** ***m*** **4** ***n*** **) trilayers. a**, Schematic of SOT switching in a magnetic TI trilayer induced by a DC pulse *I*_*x*_ under an external in-plane magnetic field μ_0_ *H*_*x*_. **b**, Circuit diagram for the current-pulse-induced SOT switching measurements. A DC pulse *I*_*x*_ with a duration of ∼ 5 ms is applied along the *x*-axis of the Hall bar device. After each pulse, the device is allowed to relax for 20 s before ρ_*yx*_ is measured using a standard AC lock-in technique with a 1 μA AC. **c-f**, *I*_*x*_-dependent ρ_*yx*_/ρ_*yx*_(0) of the (043) (**c**), (343) (**d**), (342) (**e**), and (340) (**f**) heterostructures at their respective optimal in-plane magnetic field μ_0_ *H*_x_*. The corresponding μ_0_ *H*_x_* values are ∼ 0.09 T, ∼ 0.06 T, ∼ 0.03 T, and ∼ 0.05 T for the (043), (343), (342), and (340) heterostructures, respectively. The corresponding ρ_*yx*_(0) values are ∼ 266.5 Ω, ∼ 912.9 Ω, ∼ 2.645 kΩ, and ∼ 215.7 Ω for the (043), (343), (342), and (340) heterostructures, respectively. Here, ρ_*yx*_(0) is measured after application of the DC pulse *I*_*x*_. All measurements are performed at (*V*_g_ – *V*_g_^0^) = −20 V and *T* = 2 K.

In our current-pulse-induced SOT switching measurements, a direct current (DC) pulse and an alternating current (AC) are applied in parallel to the source contact of the Hall bar device (Figure 2b). The DC pulse *I*_*x*_ with a duration of ∼ 5 ms is applied along the *x*-axis of the Hall bar device. After injecting this pulse, the device is allowed to balance for 20 s before the Hall resistance ρ_*yx*_ is measured using a standard lock-in technique with a 1 μA AC. The Hall bar devices become charged and stabilized after the first current pulse due to the property of the SrTiO_3_(111) substrate. The charging effect, induced by the DC pulse, originates from the SrTiO_3_(111) substrate (Figure S1) and is also responsible for the hysteresis in the *V*_g_-dependent transport measurements.^43^

Figure 2c-f shows the dependence of the normalized Hall resistance ρ_*yx*_/ρ_*yx*_(0) on the DC pulse *I*_*x*_ for the (043), (343), (342), and (340) heterostructures, respectively. The values of ρ_*yx*_(0) are measured after applying the DC pulse *I*_*x*_ at (*V*_g_ – *V*_g_^0^) = −20 V and under an optimal in-plane magnetic field μ_0_ *H*_x_*. Here, μ_0_ *H*_x_* is defined as the in-plane magnetic field where the switching ratio Δρ_*yx*_/ρ_*yx*_(0) reaches its maximum (Figure 3). Δρ_*yx*_ characterizes the maximum switched Hall resistance and is obtained by averaging the [ρ_*yx*_(+*I*_*x*_) – ρ_*yx*_(−*I*_*x*_)]/2 over DC pulses *I*_*x*_ > 150 μA, where the switched Hall resistance is saturated, and the switching ratio Δρ_*yx*_/ρ_*yx*_(0) measures the fraction of the Hall resistance switched by the DC pulse. ρ_*yx*_(0) is ∼ 266.5 Ω, ∼ 912.9 Ω, ∼ 2.645 kΩ, and ∼ 215.7 Ω for the (043), (343), (342), and (340) heterostructures, respectively. We find that the switching ratio Δρ_*yx*_/ρ_*yx*_(0) is ∼ 70.1% and ∼ 68.2% for the (043) and (343) heterostructures, respectively, whereas it is significantly smaller, specifically ∼ 16.0% and ∼ 28.9%, for the (342) and (340) heterostructures, respectively. We note that the SOT switching ratio of ∼ 70.1% observed in the (043) heterostructure substantially exceeds the values reported in prior studies of magnetic TI heterostructures,^21,44^ demonstrating high SOT switching efficiency in this device. Moreover, the observed reversal of the switching polarity is consistent with the SOT mechanism discussed above.

**Figure 3:**
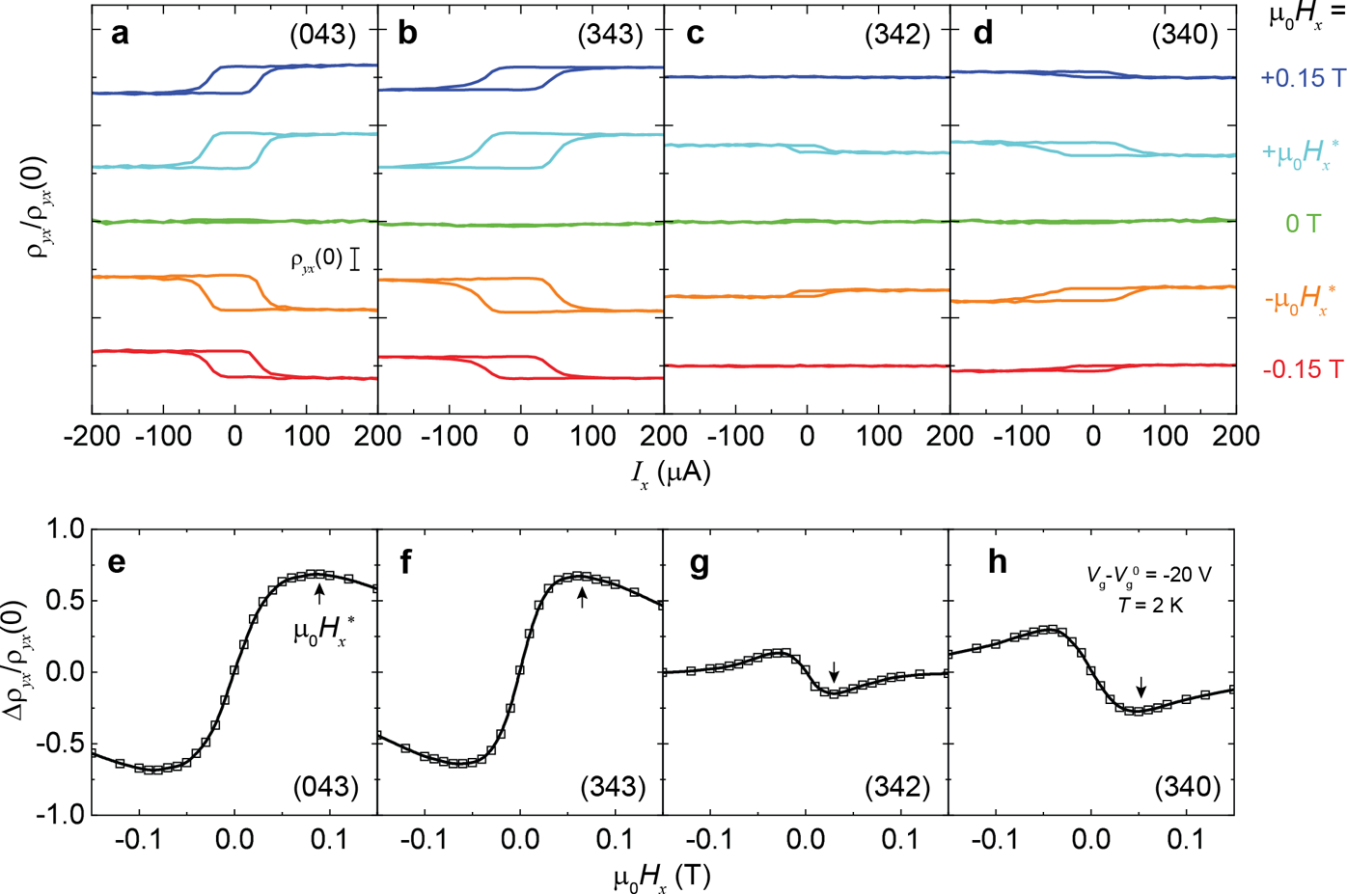
**DC-pulse-induced SOT switching in magnetic TI (** ***m*** **4** ***n*** **) trilayers under different μ**_**0**_ ***H***_***x***_ **. a-d**, *I*_*x*_-dependent ρ_*yx*_/ρ_*yx*_(0) for the (043) (**a**), (343) (**b**), (342) (**c**), and (340) (**d**) heterostructures at different μ_0_ *H*_*x*_. **e-h**, μ_0_ *H*_*x*_-dependent Δρ_*yx*_/ρ_*yx*_(0) for the (043) (**e**), (343) (**f**), (342) (**g**), and (340) (**h**) heterostructures. Black arrows indicate the optimal in-plane magnetic field μ_0_ *H*_x_*. All measurements are performed at (*V*_g_ – *V*_g_^0^) = −20 V and *T* = 2 K.

Next, we summarize the key observations. First, the switching ratio of the (043) heterostructure is significantly larger than that of the (340) heterostructure despite their purely inverted configuration. Second, the (343) heterostructure exhibits a similarly high switching ratio as the (043) heterostructure despite the top magnetic TI layer being expected to counteract the switching direction. When the bottom magnetic TI layer thickness is reduced to 2 QL in the (342) heterostructure, the switching behavior changes abruptly, with both the switching polarity and ratio resembling those of the (340) heterostructure. We attribute this observation to the ferromagnetic interlayer coupling between the top and bottom magnetic TI layers. The bottom 3 QL Cr-doped (Bi,Sb)_2_Te_3_ layer exhibits stronger switching than the top 3 QL Cr-doped (Bi,Sb)_2_Te_3_ layer. Through interlayer exchange coupling, the bottom 3 QL Cr-doped (Bi,Sb)_2_Te_3_ layer thus governs the switching ratio of the (343) heterostructure. When the thickness of the bottom Cr-doped (Bi,Sb)_2_Te_3_ layer is reduced to 2 QL, the weakened perpendicular magnetic anisotropy allows the top 3 QL Cr-doped (Bi,Sb)_2_Te_3_ layer to dominate the magnetic ordering, resulting in comparable switching ratios in the (342) and (340) heterostructures.

To further confirm that SOT drives the observed electrical switching, we measure the *I*_*x*_-dependent ρ_*yx*_/ρ_*yx*_(0) curves for the (043), (343), (342), and (340) heterostructures under different μ_0_ *H*_*x*_ (Figure 3a-d). At μ_0_ *H*_*x*_ = 0 T, none of the four heterostructures exhibits electrical switching. For the (043) and (343) heterostructures, both ρ_*yx*_ and ***M*** switch to the +*z* (−*z*) axis when *I*_*x*_ and μ_0_ *H*_*x*_ are parallel (antiparallel) (Figure 3a,b). In contrast, the electrical switching polarity is reversed in the (342) and (340) heterostructures (Figure 3c,d). We summarize the switching ratio Δρ_*yx*_/ρ_*yx*_(0) as a function of μ_0_ *H*_*x*_ for the (043), (343), (342), and (340) heterostructures (Figure 3e-h). For all four heterostructures, |Δρ_*yx*_/ρ_*yx*_(0)| first increases with increasing μ_0_ *H*_*x*_ and then decreases. The optimal in-plane magnetic field for SOT switching μ_0_ *H*_x_* is ∼ 0.09 T, ∼ 0.06 T, ∼ 0.03 T, and ∼ 0.05 T for the (043), (343), (342), and (340) heterostructures, respectively (Figure 3e-h). As noted above, an in-plane magnetic field μ_0_ *H*_*x*_ is required to create an energy difference between the ± ***M*** states and is crucial for SOT switching. A moderate μ_0_ *H*_*x*_ enhances the SOT switching ratio, whereas a large μ_0_ *H*_*x*_ tilts the magnetization into the sample plane, thereby reducing the SOT switching efficiency.

Finally, we discuss the physical origin of SOT switching in magnetic TI trilayers, focusing on the interplay between the switching behaviors of the bottom and top Cr-doped (Bi,Sb)_2_Te_3_ layers. By applying DC pulse injection during SOT switching, the magnetic TI trilayers are driven into the hole-doped regime due to the charging effect induced by the SrTiO_3_(111) substrate (Figures S1 to S3). Because the bottom Cr-doped (Bi,Sb)_2_Te_3_ layer is in direct contact with the SrTiO_3_(111) substrate, it experiences the strongest charging effect, which shifts its chemical potential *E*_F_ further downward toward the bulk valence band (Figure 4a). In contrast, the top Cr-doped (Bi,Sb)_2_Te_3_ layer is spatially separated from the SrTiO_3_(111) substrate by the middle (Bi,Sb)_2_Te_3_ spacer layer and the bottom Cr-doped (Bi,Sb)_2_Te_3_ layer, so it is partially screened from the substrate-induced electric field. The asymmetry in *E*_F_ between the top and bottom Cr-doped (Bi,Sb)_2_Te_3_ layers results in unequal spin accumulation and asymmetric SOT switching ratios (Figure 4a). Because the charging effect during DC pulse injection is difficult to characterize directly, we investigate the *E*_F_ asymmetry through electrostatic gating and transport measurements. The asymmetric chemical potential is further corroborated by the μ_0_ *H*_⊥_-dependent ρ_*yx*_ curves of the (043) and (340) heterostructures at (*V*_g_ – *V*_g_^0^) = 0 V and ± 100 V (Figure 4b,c). For (*V*_g_ – *V*_g_^0^) = −100 V, i.e., the *p*-doped regime, the ferromagnetism is enhanced, whereas for (*V*_g_ – *V*_g_^0^) = +100 V, i.e., the *n*-doped regime, the ferromagnetism is weakened.^45^ Figure 4d,e shows the (*V*_g_ – *V*_g_^0^) dependence of the coercive field μ_0_ *H*_c_. Although both the (043) and (340) heterostructures exhibit similar trends, μ_0_ *H*_c_ saturates at ∼ 0.086 T for the (043) heterostructure, compared with only ∼ 0.055 T for the (340) heterostructure. Because the Hall hysteresis loops at (*V*_g_ – *V*_g_^0^) = 0 V are nearly identical, the observed asymmetry cannot be attributed to MBE growth-induced variations reported in a prior study of (Bi,Sb)_2_Te_3_ films on InP(111) substrates.^46^ These results indicate that the bottom Cr-doped (Bi,Sb)_2_Te_3_ layer is more strongly charged than the top Cr-doped (Bi,Sb)_2_Te_3_ layer, resulting in larger spin accumulation and an enhanced SOT switching ratio.

**Figure 4:**
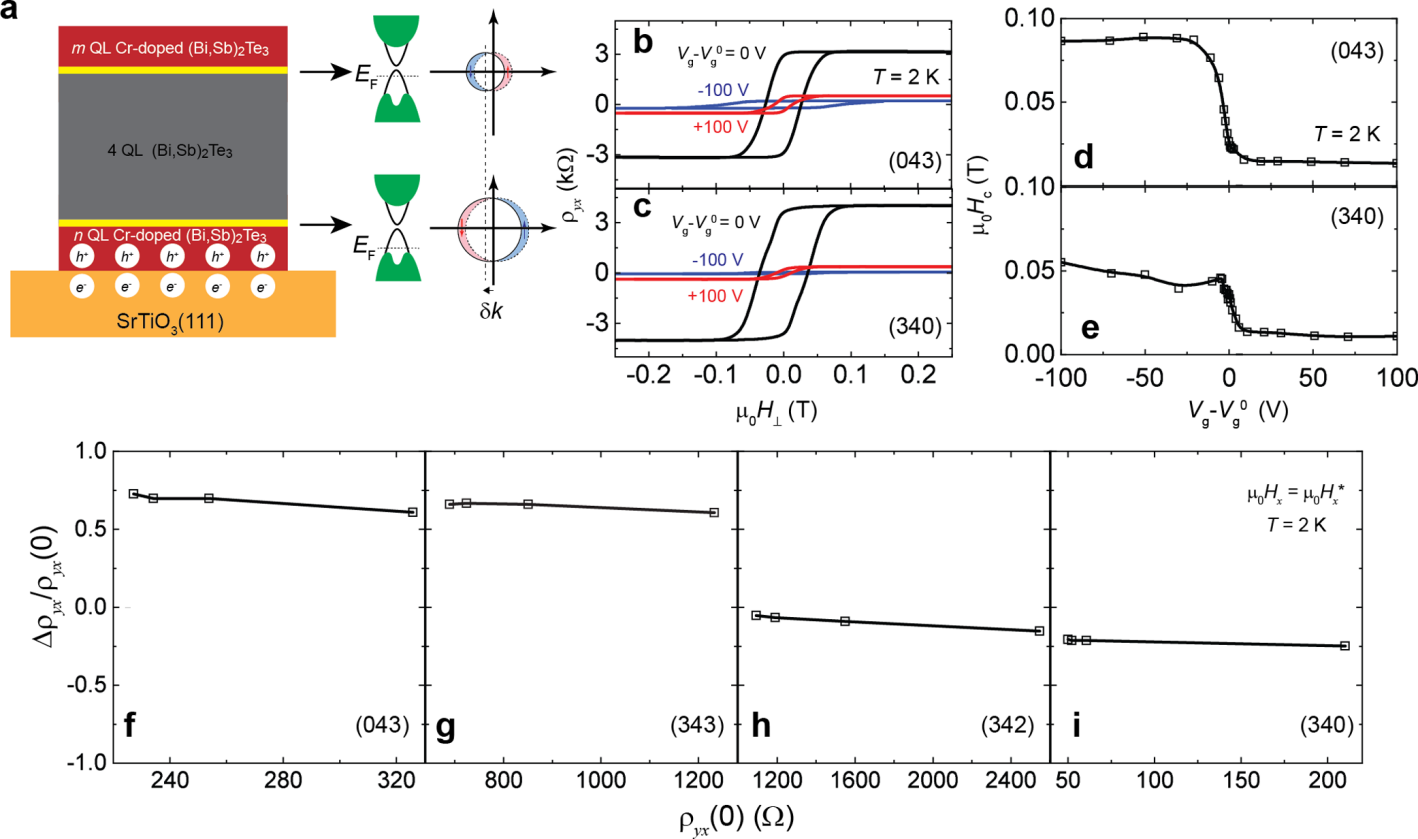
**Origin of SOT switching in magnetic TI (** ***m*** **4** ***n*** **) trilayers. a**, Left: Schematic of the charging effect induced by the SrTiO_3_(111) substrate. Right: Resulting asymmetric chemical potential *E*_F_ and spin accumulation during DC pulse injection. The green region is the bulk band, and the parabolic curves are the gapped topological surface states. **b,c**, μ_0_ *H*_⊥_-dependent ρ_*yx*_ measured at (*V*_g_ – *V*_g_^0^) = 0 V and ± 100 V for the (043) (**b**) and (340) (**c**) heterostructures. **d, e**, (*V*_g_ – *V*_g_^0^) dependence of μ_0_ *H*_c_ for the (043) (**d**) and (340) (**e**) heterostructures. **f-i**, Δρ_*yx*_/ρ_*yx*_(0) plotted as a function of ρ_*yx*_(0) for the (043) (**f**), (343) (**g**), (342) (**h**), and (340) (**i**) heterostructures.

To further examine how the *E*_F_ asymmetry affects the SOT switching ratio, we perform current-pulse-induced SOT switching measurements on the (043), (343), (342), and (340) heterostructures at different *E*_F_ (Figure 4f-i). A smaller ρ_*yx*_(0) value corresponds to stronger hole doping, indicating a lower *E*_F_. We note that all four heterostructures consistently charge toward the hole-doped side after the first DC pulse and remain stable thereafter, even when initially tuned to the charge-neutral point or electron-doped regime. This current pulse-induced charging effect is absent in our heterostructures grown on InP(111)­A substrates, suggesting that it originates from the complex dynamic response of the SrTiO_3_(111) substrate (Figure S3).

With increasing hole doping, the switching ratio increases from ∼ 60.7% to ∼ 72.7% for the (043) heterostructure and from ∼ 60.6% to ∼ 66.0% for the (343) heterostructure (Figure 4f,g). In contrast, the (342) and (340) heterostructures, which exhibit opposite switching polarity, show reduced SOT switching ratios, from 15.3% to 5.4% for the (342) heterostructure and from 24.9% to 20.6% for the (340) heterostructure (Figure 4h, i). These observations can be understood as follows: when *E*_F_ moves away from the charge-neutral point, i.e., (*V*_g_ – *V*_g_^0^) = 0 V, more holes accumulate on the bottom Cr-doped (Bi,Sb)_2_Te_3_ layer than on the top, thereby strengthening the SOT switching from the bottom Cr-doped (Bi,Sb)_2_Te_3_ layer. For the (043) and (343) heterostructures, the overall SOT is enhanced, leading to an increase in the SOT switching ratio. In contrast, for the (342) heterostructure, the enhanced SOT from the bottom Cr-doped (Bi,Sb)_2_Te_3_ layer counteracts that from the top Cr-doped (Bi,Sb)_2_Te_3_ layer, leading to a reduced SOT switching ratio. For the (340) heterostructure, the more substantial hole accumulation at the bottom Cr-doped (Bi,Sb)_2_Te_3_ layer directs more current through the bottom gapless surface states, thereby diminishing the SOT switching efficiency of the top Cr-doped (Bi,Sb)_2_Te_3_ layer (Figure 1c). Therefore, our results demonstrate that the SrTiO_3_(111) substrate-induced asymmetry in *E*_F_ significantly enhances the SOT switching ratio in the bottom Cr-doped (Bi,Sb)_2_Te_3_ layer, enabling electrical switching of edge-current chirality in QAH trilayers (Figure S2).^6^ Moreover, the 2D interfacial charge-to-spin conversion efficiency θ_*CS*_^2*D*^ in the (343) heterostructure is estimated to be ∼ 0.05 nm^–1^ and reaches its maximum at *V*_g_ = *V*_g_^0^ (ref 6). This behavior confirms that the enhanced SOT switching ratio in the heavily hole-doped regime originates from the increased *E*_F_ asymmetry rather than from an enhancement of θ_*CS*_^2*D*^.

To summarize, we use electron-beam lithography to fabricate a series of magnetic TI trilayer Hall bar devices and demonstrate that SOT is an efficient means to manipulate both magnetization and edge current chirality in QAH insulators. We find that the large SOT switching ratio observed in QAH trilayers originates from the SrTiO_3_(111) substrate-induced *E*_F_ asymmetry, establishing the foundation for the electrical control of edge-current chirality in QAH insulators. These findings advance our understanding of SOT-driven magnetization switching in magnetic TI multilayers and pave the way for the development of next-generation energy-efficient QAH-based electronic and spintronic devices.

## Supplementary Material



## Data Availability

The data that support the findings of this article are openly available at Zenodo (10.5281/zenodo.19478382).

## Notes

MBE growth of magnetic TI trilayers, electron-beam lithography of the magnetic TI Hall bar devices, electrical transport measurements, more discussion on the SrTiO_3_(111) substrate-induced charging effect and electrical switching of edge-current chirality in QAH insulators, and more transport results of magnetic TI trilayers (PDF)
