# Supplementary material for: Designing Magnetic Topological Insulator Trilayers for Highly Efficient Spin–Orbit Torque Switching
Source: Nano Lett. 2026 Jun 4;26(23):7833–40. doi: 10.1021/acs.nanolett.6c02154 (PMC13281699; doi:10.1021/acs.nanolett.6c02154)
Supplement: Supplementary file 1 [file nl6c02154_si_001.pdf]

Supporting Information

Designing Magnetic Topological Insulator Trilayers for Highly-Efficient Spin-Orbit Torque Switching

Ling-Jie Zhou[1], Deyi Zhuo[1], Han Tay[1], Zi-Jie Yan[1], Pu Xiao[1], Xiaoda Liu[1], Bomin Zhang[1], and Cui-Zu Chang[1]

[1] Department of Physics, The Pennsylvania State University, University Park, PA 16802, USA

Corresponding authors: cxc955@psu.edu (C.-Z. C.).

Contents:

I. Supplementary Text

1. Experimental methods

2. Discussion on the $SrTiO_3$ substrate-induced charging effect

3. Electrical switching of edge-current chirality in QAH insulators

4. Pathways towards achieving a 100% SOT switching ratio in magnetic TI trilayers

5. Discussion on thermal effect in SOT switching

II. Supplementary Figures

References

I. Supplementary Text

1. Experimental methods

1.1 MBE growth

The magnetic TI samples used in this work are *m* QL $(Bi,Sb)_{1.73}Cr_{0.27}Te_3$/4 QL $(Bi,Sb)_2Te_3$/*n* QL $(Bi,Sb)_{1.73}Cr_{0.27}Te_3$ [denoted as (*m*4*n*)] grown on heat-treated $SrTiO_3$(111) and InP(111)A substrates. The insulating $SrTiO_3$(111) substrates are first soaked in 90°C deionized water for 1.5 hours and then annealed at 979 °C for 3 hours in a tube furnace with flowing high-purity O_2. After this heat treatment, the $SrTiO_3$(111) substrate exhibits a single TiO_2-terminated surface that is passivated and atomically flat. All magnetic TI heterostructures are synthesized in a commercial MBE chamber (Omicron Lab10) with a base pressure below 2×10^{-10} mbar. Before growth, the 0.5 mm-thick insulating $SrTiO_3$ (111) substrates are outgassed at T = 600 °C for 1 hour, while the 0.35 mm-thick insulating InP(111)A substrates are outgassed at T = 300°C for 1 hour. High-purity Bi (99.9999%), Sb (99.9999%), Cr (99.999%), and Te (99.9999%) are evaporated from Knudsen effusion cells. During growth, the substrate is maintained at ~230 °C. The Te/(Bi + Sb + Cr) flux ratio is set to exceed 10 to prevent Te deficiency in the samples. The growth rate for the magnetic TI heterostructure samples is ~0.2 QL/min. To avoid contamination and degradation under ambient conditions, a 3 nm-thick Al_2O_3 layer is deposited on the magnetic TI heterostructures using atomic layer deposition (ALD) immediately after they are removed from the MBE chamber.

1.2 Electron-beam lithography of the magnetic TI Hall bar devices

The magnetic TI Hall bar devices, with a width *w* of 2 μm, are fabricated using a two-step electron-beam lithography process. The Hall bar devices have an aspect ratio of ~4. A Cr (10nm)/Au (30nm) layer is used as the electrodes. Before the deposition of the Cr/Au electrodes by electron-beam evaporation, the 3 nm Al_2O_3 capping layer is etched using CD26. The magnetic

TI heterostructures are then patterned into a Hall-bar geometry by Ar plasma etching. To minimize charging during electron-beam lithography, a 20 nm Au layer is deposited on the resist beforehand. For the (343) Hall bar devices on InP(111)A substrates, a 30 nm Al_2O_3 layer deposited by ALD is used as the gate dielectric after the two-step electron-beam lithography process. The top-gate electrode is then fabricated in a third electron-beam lithography, followed by deposition of the Cr (10nm)/Au (30nm) layer by electron-beam evaporator and a subsequent liftoff process.

1.3 Electrical transport measurements

Electrical contacts for transport measurements are made by pressing indium dots on the Hall bar devices. The bottom-gate electrode is prepared by flattening indium spheres on the backside of the $SrTiO_3$ substrate. Electrical transport measurements are carried out using a Physical Property Measurement System (PPMS) with a rotator (Quantum Design DynaCool, 1.7 K, 9 T). The gate voltage V_g is applied using a Keithley 2450 source meter. An AC excitation current of 1 μA is used. DC pulses with a duration of 5 milliseconds are applied using a Keithley 6221 AC/DC source. The longitudinal and Hall resistances are measured with an SR860 lock-in amplifier (Stanford Research Systems). All magnetotransport results shown in this work are symmetrized or anti-symmetrized as a function of the magnetic field to remove contributions from electrode misalignments.

2. Discussion on the $SrTiO_3$ substrate-induced charging effect

As noted in the main text, the DC pulse injection can charge the magnetic TI heterostructures. Before the DC pulse injection measurement, the (343) heterostructure is tuned to the charge neutral point, i.e., $V_g = V_g^0 = +5$ V. After magnetic field training, $\rho_{yx}(0)$ is ~22.9 kΩ (Fig. S1a). Next, we perform the DC pulse injection measurement, and the final state is marked by a star (Fig. S1b). After the DC pulse injection measurement, the $\mu_0 H$ dependence of ρ_{xx} and ρ_{yx} is shown in Fig. S1c.

After magnetic field training, $\rho_{yx}(0)$ is reduced to only ~1.8 kΩ. Because the back gate voltage V_g is kept at V_g = +5 V throughout the entire measurement, so the charging effect must be induced by the $SrTiO_3$ substrate during the DC pulse injection measurement.

To further verify the $SrTiO_3$ substrate-induced charging effect, we prepare a control sample, 3 QL $(Bi,Sb)_{1.73}Cr_{0.27}Te_3$/4QL $(Bi,Sb)_2Te_3$/3 QL $(Bi,Sb)_{1.73}Cr_{0.27}Te_3$ grown on InP(111)A substrate with a top gate. The top-gate dielectric is Al_2O_3. Figure S3a shows μ_0H-dependent ρ_{xx} and ρ_{yx} of the (343) heterostructure on InP(111)A measured at $V_g = V_g^0$ = +3 V. $\rho_{yx}(0)$ is found to be ~21.2 kΩ. Figure S3b shows I_x-dependent ρ_{yx} for the (343) heterostructure on InP(111)A, with the final state marked by a star. After the DC pulse injection measurement, the sample is magnetized by an out-of-plane magnetic field. After magnetic field training, $\rho_{yx}(0)$ remains ~20.8 kΩ, indicating a negligible charging effect (Fig. S3c). Therefore, we conclude that the charging effect originates from the $SrTiO_3$(111) substrate. We attribute the charging effect primarily to the quantum paraelectric nature of $SrTiO_3$(111) (Ref.[1,2]). As T decreases from 300 K to 2 K, the dielectric constant ε increases from ~ 300 to ~30000 (Ref.[3]). In addition, a hysteresis loop has been observed in the dielectric constant ε as a function of applied voltage, which has been attributed to the presence of oxygen vacancies in $SrTiO_3$ (Ref.[1]). During the DC pulse measurements, the sample charges and relaxes rapidly, so the dielectric constant ε may not fully recover to its pre-pulse value, giving rise to the observed charging effect. Related hysteretic behavior has also been observed in gate-sweep measurements [4,5].

3. Electrical switching of edge-current chirality in QAH insulators

In the QAH devices, the chirality of the edge current is determined by the magnetization direction. Figure S2 shows that that the edge can be through-SOT-induced magnetization reversal

switches the current chirality. The (343) heterostructure is initially magnetized into the C = +1 QAH state at $V_g = V_g^0$, with $\rho_{yx}(0) \approx 22.9$ kΩ. The DC pulse injection measurement is then performed to switch the magnetization (Fig. S2a). No out-of-plane magnetic field is applied, and the magnetization reversal is driven entirely by SOT, confirmed by the nearly equal and opposite values of ρ_{yx} in the positive and negative saturation regimes, i.e., $|I_x| > 150$ μA. However, because of the $SrTiO_3$-induced charging effect, the (343) heterostructure is tuned hole-doped and far from the charge-neutral point. To discharge the $SrTiO_3$ substrate, we first sweep V_g to -100 V and then to +100 V. The minimum ρ_{yx} reaches -16.3 kΩ, accompanied by a dip in ρ_{xx}, indicating that the (343) heterostructure is switched into the C = -1 QAH state (Fig. S2b). We note that perfect quantization of ρ_{yx} and vanishing ρ_{xx} can be achieved by measuring the (343) heterostructure at T = 25mK (ref.[6]).

4. Pathways towards achieving a 100% SOT switching ratio in magnetic TI trilayers

To achieve a 100% SOT switching ratio in magnetic TI bilayers or trilayers, several possible strategies can be considered. First, chemical potential optimization may further improve the SOT switching ratio. As noted in our manuscript, the SOT switching ratio is governed by the chemical potential asymmetry between the top and bottom Cr-doped $(Bi,Sb)_2Te_3/(Bi,Sb)_2Te_3$ interfaces. Further engineering of this chemical potential asymmetry through dual-gate electrical control could maximize the differential spin accumulation at the two $(Bi,Sb)_2Te_3/(Bi,Sb)_2Te_3$ interfaces and potentially increase the SOT switching ratio. Second, substrate engineering could provide an additional route. As noted in our manuscript, since the $SrTiO_3(111)$-induced charging effect plays a key role in generating the chemical potential asymmetry, using substrates with stronger or more controllable dielectric response could further enhance the SOT switching ratio. Third, device miniaturization may also be beneficial. If the lateral dimensions of the device are reduced to the

single domain limit, 100% SOT switching may become possible because the device would have only two well-defined magnetic states, without averaging over multiple magnetic domains.

5. Discussion on thermal effect in SOT switching

In our experiments, thermal effect does play a role in the SOT switching of magnetic TI bilayer and trilayers. However, the heating effect is local and is not detectable through the thermometer (i.e., the reading temperature of the PPMS). The sample relaxes to a stable state within 1s after injecting the current pulse. The 20s waiting time is primarily used to allow the lock-in amplifier to stabilize and provide a reliable reading. The heating effect during SOT switching of magnetic TI trilayers has been discussed in our recent work [6]. The instantaneous temperature is estimated to be ~ 20 K, which is lower than the Curie temperature of the sample (~ 35 K). The thermal effect likely reduces the magnetic anisotropy and thus assists the SOT switching. To generate a chemical potential difference through the Seebeck effect, a temperature gradient is required. However, for all samples used in this work, the thickness is less than 10 nm, and the top and bottom surfaces should be well thermalized, with a negligible temperature difference. Therefore, as noted in our manuscript, we believe the chemical potential asymmetry originates from the quantum paraelectric properties of the $SrTiO_3$(111) substrate, rather than the current-induced heating effect.

II. Supplementary Figures

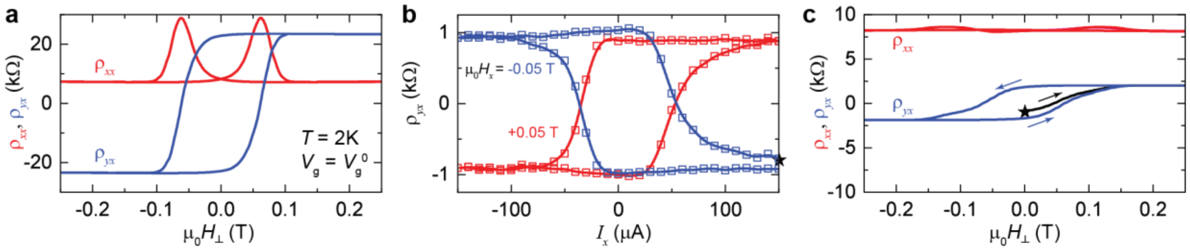

Fig. S1| $SrTiO_3$ substrate-induced charging effect. a, $\mu_0 H$-dependent ρ_{xx} and ρ_{yx} of the (343) heterostructure measured at V_g = +5 V and T = 2 K. **b,** I_x-dependent ρ_{yx} for the (343) heterostructure measured at $\mu_0 H_x$ = +0.05 T (red) and $\mu_0 H_x$ = -0.05 T (blue).Hall resistance ρ_{yx} as a function of I_x under opposite in-plane magnetic field $\mu_0 H_x$. The final state is marked by a star. **c,** $\mu_0 H$-dependent ρ_{xx} and ρ_{yx} after the DC-pulse injection measurement. The post-pulse state is marked by a star, and the magnetic-field-sweep direction is indicated by arrows. $\rho_{yx}(0)$ decreases from ~22.9 kΩ to ~1.8 kΩ, indicating a strong charging effect induced by DC-pulse injection. All measurements are performed at V_g = +5 V and T = 2 K.

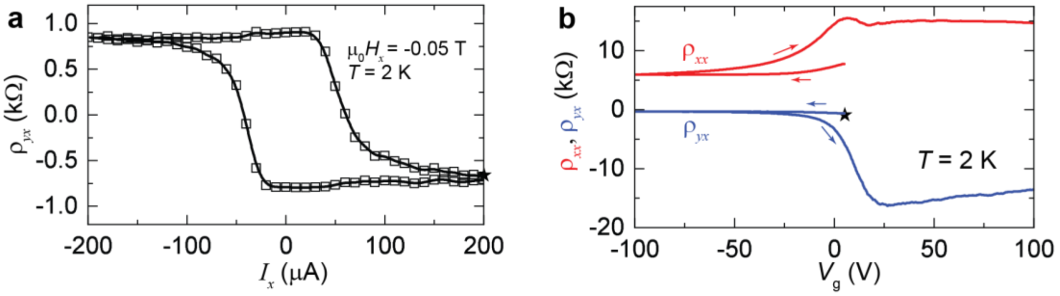

Fig. S2| Electrical switching of edge-current chirality through SOT. a, I_x-dependent ρ_{yx} of the (343) heterostructure measured at $\mu_0 H_x$ = -0.05 T and V_g = +5 V. The final state is marked by a star. The sample is initially in the C = +1 QAH state before the DC pulse injection measurement, with ρ_{yx} = +22.9 kΩ. **b,** V_g-dependent ρ_{xx} and ρ_{yx} of the (343) heterostructure measured without an external magnetic field. The post-pulse state is marked by a star, and the V_g-sweep direction is indicated by arrows. To compensate for the charging effect, the $SrTiO_3$ is first discharged to -100 V, then tuned to +100 V. The minimum $\rho_{yx}(0)$ reaches -16.3 kΩ, accompanied by a dip in ρ_{xx}, indicating that the (343) heterostructure switches to C = -1 QAH state. All the measurements are performed at T = 2 K.

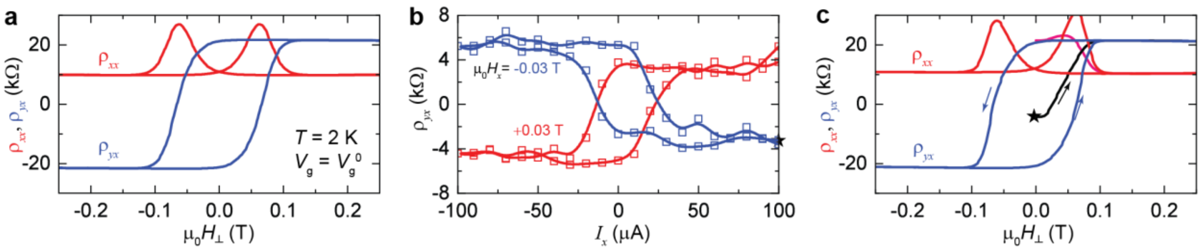

Fig. S3| Absence of the charging effect in a magnetic TI heterostructure grown on an InP(111)A substrate. a, $\mu_0 H$-dependent ρ_{xx} and ρ_{yx} of the (343) heterostructure measured at $V_g = V_g^0 = +3$ V. **b,** I_x-dependent ρ_{yx} for the (343) heterostructure measured at $\mu_0 H_x = +0.03$ T (red) and $\mu_0 H_x = -0.03$ T (blue). The final state is marked by a star. ρ_{yx} as a function of I_x under opposite in-plane magnetic field $\mu_0 H_x$. **c,** $\mu_0 H$-dependent ρ_{xx} and ρ_{yx} after the DC-pulse injection measurement. The post-pulse state is marked by a star, and the magnetic-field-sweep direction is indicated by arrows. After magnetic field training, $\rho_{yx}(0)$ remains ~20.8 kΩ, , indicating a negligible charging effect. All the measurements are performed at $V_g = +3$ V and $T = 2$ K.

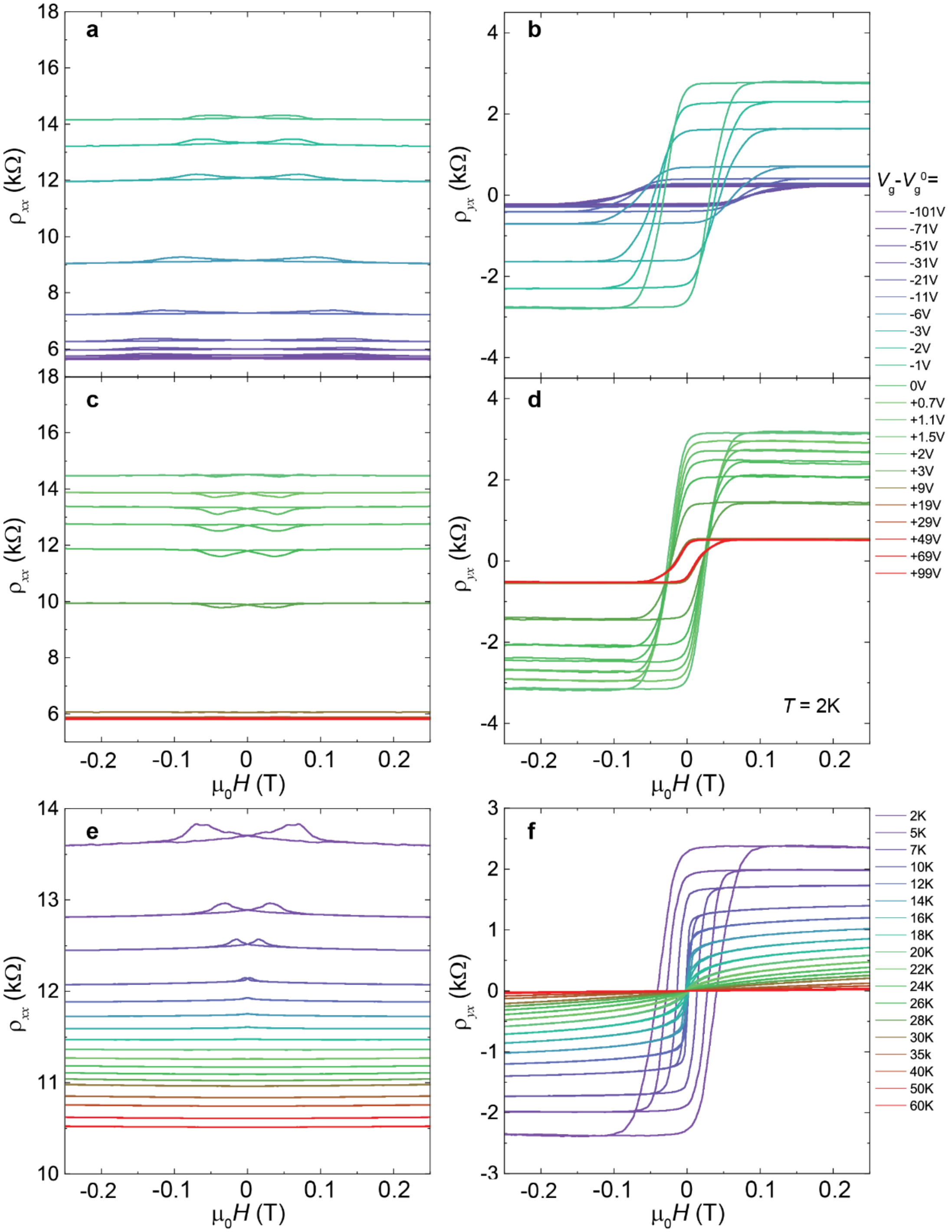

Fig. S4| More transport results of the (043) heterostructure. a, c, $\mu_0 H$ dependence of ρ_{xx} measured at different V_g. **b, d,** $\mu_0 H$ dependence of ρ_{yx} measured at different V_g. **e, f,** $\mu_0 H$-dependent ρ_{xx} (**e**) and ρ_{yx} (**f**) measured at different T. All measurements in (**a-d**) are performed at $T = 2$ K. All measurements in (**e, f**) are performed at $V_g = V_g^0 = +1$ V.

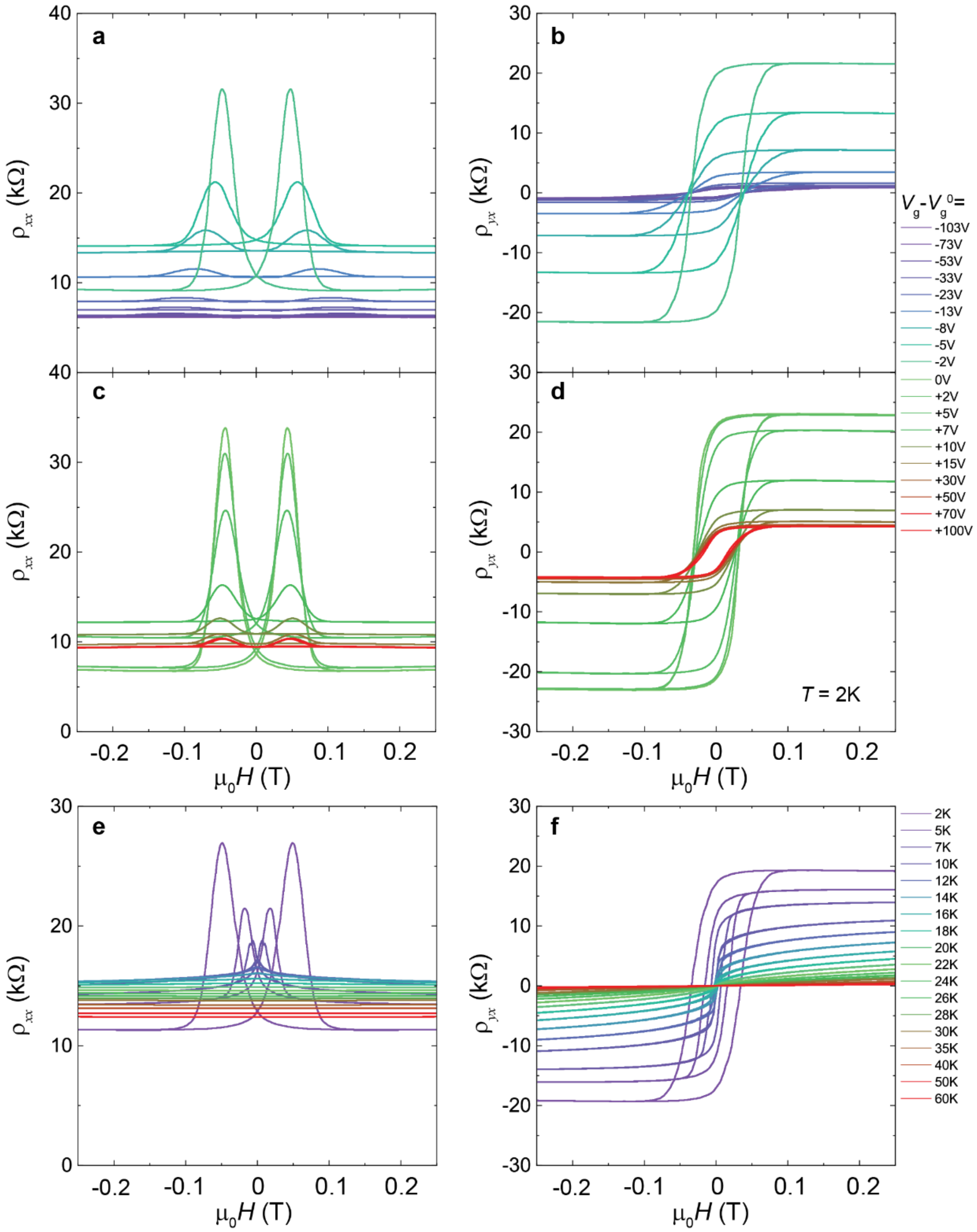

Fig. S5| More transport results of the (343) heterostructure. Same as Fig. S4, but for the (343) heterostructure. All measurements in (**e, f**) are performed at $V_g = V_g^0 = +5$ V.

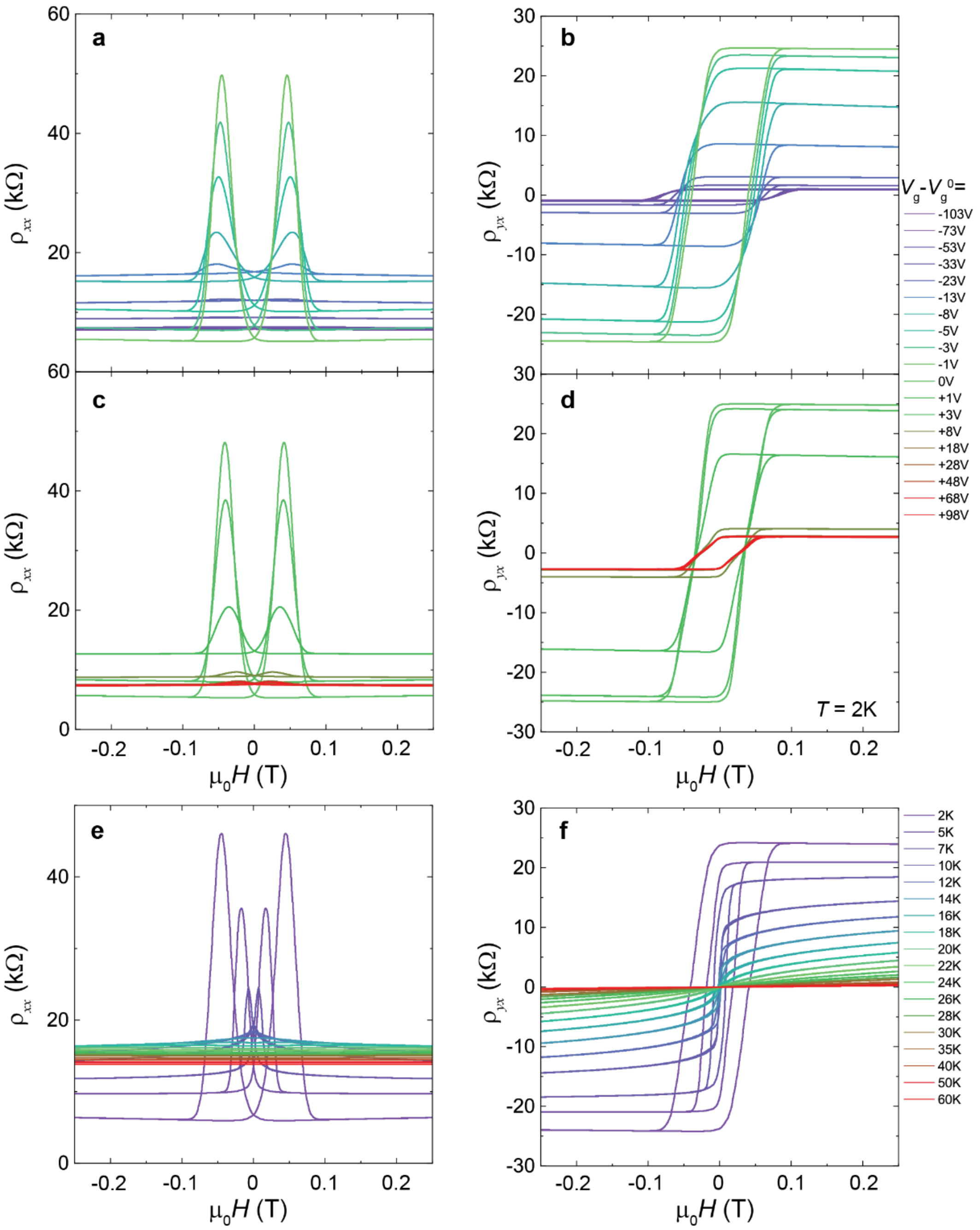

Fig. S6| More transport results of the (342) heterostructure. Same as Fig. S4, but for the (342) heterostructure. All measurements in (**e, f**) are performed at $V_g = V_g^0 = +2$ V.

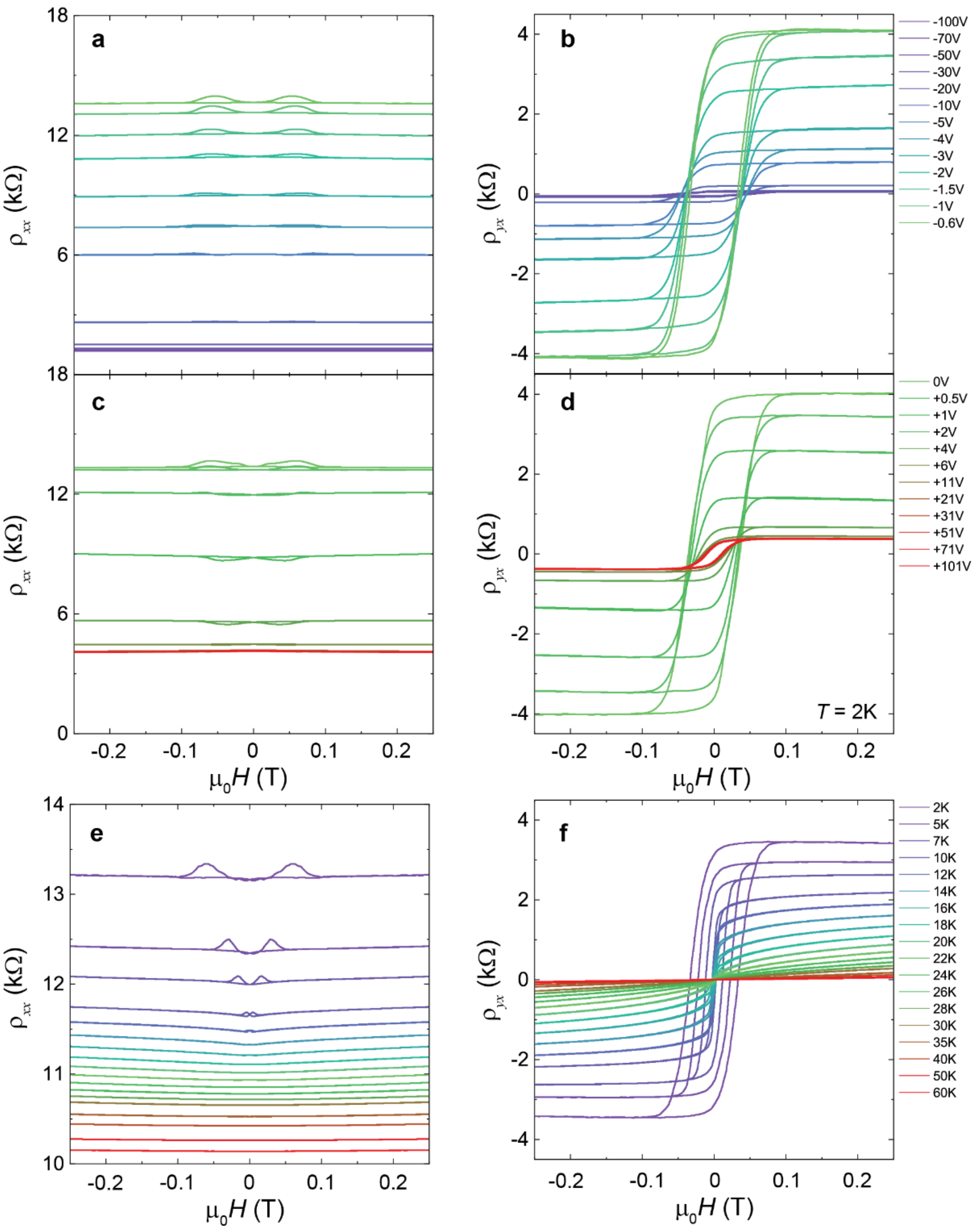

Fig. S7| More transport results of the (340) heterostructure. Same as Fig. S4, but for the (340) heterostructure. All measurements in (**e, f**) are performed at $V_g = V_g^0 = -1$ V.

References

1. Davidovikj, D.; Manca, N.; van der Zant, H. S. J.; Caviglia, A. D.; Steele, G. A. Quantum paraelectricity probed by superconducting resonators. *Phys. Rev. B* **2017**, 95, 214513.
2. Müller, K. A.; Burkard, H. $SrTiO_3$: An intrinsic quantum paraelectric below 4 K. *Phys. Rev. B* **1979**, 19, 3593.
3. Sakudo, T.; Unoki, H. Dielectric Properties of $SrTiO_3$ at Low Temperatures. *Phys. Rev. Lett.* **1971**, 26, 851.
4. Chang, C.-Z.; Zhao, W.; Li, J.; Jain, J. K.; Liu, C.; Moodera, J. S.; Chan, M. H. W. Observation of the Quantum Anomalous Hall Insulator to Anderson Insulator Quantum Phase Transition and its Scaling Behavior. *Phys. Rev. Lett.* **2016**, 117, 126802.
5. Sachs, R.; Lin, Z.; Shi, J. Ferroelectric-like $SrTiO_3$ surface dipoles probed by graphene. *Sci. Rep.* **2014**, 4, 3657.
6. Yuan, W.; Zhou, L.-J.; Yang, K.; Zhao, Y.-F.; Zhang, R.; Yan, Z.; Zhuo, D.; Mei, R.; Wang, Y.; Yi, H.; Chan, M. H. W.; Kayyalha, M.; Liu, C.-X.; Chang, C.-Z. Electrical switching of the edge current chirality in quantum anomalous Hall insulators. *Nat. Mater.* **2024**, 23, 58.
